# Prediction of Hemodynamic-Related Hemolysis in Carotid Stenosis and Aiding in Treatment Planning and Risk Stratification Using Computational Fluid Dynamics

**DOI:** 10.3390/biomedicines12010037

**Published:** 2023-12-22

**Authors:** Krystian Jędrzejczak, Wojciech Orciuch, Krzysztof Wojtas, Michał Kozłowski, Piotr Piasecki, Jerzy Narloch, Marek Wierzbicki, Łukasz Makowski

**Affiliations:** 1 Faculty of Chemical and Process Engineering, Warsaw University of Technology, Waryńskiego 1, 00-645 Warsaw, Poland; 2 Department of Cardiology and Structural Heart Diseases, Medical University of Silesia, Ziołowa 47, 40-635 Katowice, Poland; 3 Interventional Radiology Department, Military Institute of Medicine—National Research Institute, Szaserów 128, 04-141 Warsaw, Poland

**Keywords:** arteriosclerosis, hemolysis, blood, CFD, coronary artery, carotid artery, percutaneous coronary intervention, hemoglobin

## Abstract

Atherosclerosis affects human health in many ways, leading to disability or premature death due to ischemic heart disease, stroke, or limb ischemia. Poststenotic blood flow disruption may also play an essential role in artery wall impairment linked with hemolysis related to shear stress. The maximum shear stress in the atherosclerotic plaque area is the main parameter determining hemolysis risk. In our work, a 3D internal carotid artery model was built from CT scans performed on patients qualified for percutaneous angioplasty due to its symptomatic stenosis. The obtained stenosis geometries were used to conduct a series of computer simulations to identify critical parameters corresponding to the increase in shear stress in the arteries. Stenosis shape parameters responsible for the increase in shear stress were determined. The effect of changes in the carotid artery size, length, and degree of narrowing on the change in maximum shear stress was demonstrated. Then, a correlation for the quick initial diagnosis of atherosclerotic stenoses regarding the risk of hemolysis was developed. The developed relationship for rapid hemolysis risk assessment uses information from typical non-invasive tests for treated patients. Practical guidelines have been developed regarding which stenosis shape parameters pose a risk of hemolysis, which may be adapted in medical practice.

## 1. Introduction

Despite the continuous development of medicine, modern society still suffers from cardiovascular diseases [1,2,3]. Today’s lifestyle, combined with a lack of physical activity and an unhealthy diet, often leads to hypertension or atherosclerosis [4,5]. Atherosclerosis is responsible for cardiovascular diseases leading to stroke or infarction. The increase in cholesterol deposits causes a decrease in the surface area perpendicular to the flow direction, which in turn causes a local increase in shear stresses. An increase in shear stress can destroy erythrocytes [6,7,8]. This local hemolysis causes impairment of the endothelium and smooth muscle cells related to NO decrease and direct toxicity of hemoglobin and Fe^++^ released from erythrocytes [9]. Endothelial abrasion may expose deeper layers of the arterial wall rich with collagen to circulated platelets and may lead to its activation and clot formation. Hemolysis occurs in atherosclerosis from very early to advanced stages of artery stenosis [9]. The basis of atherosclerosis treatment is to cease this pathological process as soon as possible. Guidelines related to endovascular interventions in cardiovascular diseases are focused mainly on the severity of artery stenosis before angioplasty (i.e., carotid internal artery or coronal arteries). One of the most common treatment methods for patients with advanced carotid or coronary atherosclerosis is percutaneous endovascular intervention with balloon angioplasty followed by stent placement. A better understanding of atherosclerosis hemolysis etiopathology may help to establish a new guideline with interventions performed at an earlier stage of the disease. Due to the potential risk of complications, it is worth having information about the impact of the narrowing on the hydrodynamics of blood flow in the vessel. Hence, the idea is to use non-invasive blood flow measurements combined with advanced imaging and computational fluid dynamics (CFD) for a non-invasive diagnosis of atherosclerosis [10]. Appropriate diagnosis of atherosclerotic complications using CFD requires attention to blood rheology, which is a non-Newtonian fluid [11,12,13,14,15,16,17] due to the complex structure of blood. Due to the non-Newtonian nature of blood, a blood rheology model based on population balance [8] was used, which reconstructs the physiological behavior of blood related to erythrocyte agglomeration and deagglomeration depending on the shear rate. In addition to blood rheology, selecting an appropriate hemolysis model is important [6,7,18,19,20,21,22,23,24,25]. The blood rheology model based on the erythrocyte population balance has a built-in blood hemolysis model that allows for the use of information obtained from solving the size distribution of red blood cell agglomerates and the number of single erythrocytes [8]. In addition, it is crucial to select an appropriate flow turbulence model that would be useful both in terms of laminar and transient flow observed in the zone after the stenosis. Printed channel models and micro particle image velocimetry (µPIV) [10,26] were used to validate the turbulence model used in the CFD simulations. The simulations were carried out for steady flows because the difference between the values of average wall shear stresses (WSSs) for average flow and time-averaged wall shear stresses (TAWSs) for pulsating flow is minimal [27] for stenosed arteries, as shown in one of the previous articles. The analysis of the influence of the shape of the blood vessel on the risk of hemolysis was started in the last article [10], where the focus was on the degree of stenosis of the vessel and its eccentricity. As part of this work, the analysis was extended to include variable vessel size, stenosis length, and variable lumen shape in the narrowest part of the stenosis.

In this article, we wanted to address the research gap in the field of rapid initial diagnosis of atherosclerotic complications regarding the risk of blood hemolysis in the space of cholesterol constrictions. The aim of the study was to identify key parameters of the shape of the atherosclerotic stenosis that influence the increase in shear stress to which red blood cells are exposed. The universal relationship between shear stresses and the geometry of the constriction fits into modern trends in personalized medicine, while increasing the efficiency of the health care system in the case of a growing number of patients.

## 2. Materials and Methods

### 2.1. Blood Rheology and Hemolysis Model

The blood viscosity model based on population balance was presented in detail in various publications [8,10,27]. The model assumes agglomeration of red blood cells in areas with low shear rates and deagglomerations in regions with high shear rates. As a result, a physiological distribution of the size of red blood cell agglomerates can be observed across the cross-section of the vessel so that the viscosity is greatest in the axis of the vessel. In addition, thixotropic effects of blood [28,29,30,31] related to the finite rate of deagglomeration and agglomeration of erythrocytes are observed. The hemolysis model originates from power law formulation and later linearization [6,7,32]. However, population-balance-based rheology’s (PBBR’s) model refers to hemolysis to only fully deagglomerated single red blood cells, whose concentration is known thanks to the solution of population balance [8].

(1) $$\frac{\Delta H_{b}}{H_{b}}=\frac{H\cdot m_{s}L_{0}^{3}\;}{m_{s}L_{0}^{3}+\sum_{i=1}^{3}w_{i}L_{i}^{3}}.\;$$

where $m_{s}$ is the concentration of single red blood cells in direct quadrature method of moments (DQMOM), $L_{i}$ is the sizes of agglomerates used in DQMOM, and $w_{i}$ is the weights used in DQMOM.

### 2.2. Numerical Settings

The calculations in this study were performed using ANSYS Fluent 2022 R2. Model implementation was presented in detail in the previous article [8]. Similar to previous studies, the flow is mainly laminar; however, the turbulent flow is promoted by the rapid increase in velocity in the stenosis in the case of large flows [10]. The GEKO model with the transition flow option was chosen to capture both laminar and turbulent regions. The simulations were conducted for blood flow rates in the 0.625–6.25 mL/s range, similar to the previous article [10]. Simulations for different polyhedral mesh densities were performed to determine the final meshes for which the obtained results were independent of mesh size and quality. 

The following boundary conditions were applied. At the INLET, the plug flow inlet was located at an appropriate distance from the stenosis to establish the fully developed velocity profile. Inlet conditions for the population balance were calculated, assuming that the hematocrit equaled 45%. It was also assumed that no hemolyzed red blood cells were at the inlet. In the case of WALLS, the no-slip condition and homogeneous Neumann conditions for user-defined scalars were used. At the OUTLET, the atmospheric pressure and homogeneous Neumann conditions for user-defined scalars were chosen. 

## 3. Results 

### 3.1. CFD Results

**Figure 1:**
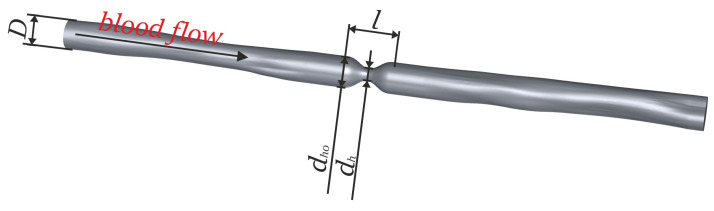
Geometry of an artery with stenosis.

The CFD analysis was carried out for an inlet velocity of 0.05 to 0.5 m/s similarly to the previous article. Within the framework of this article, the range of analyzed geometries of cholesteric stenoses was extended by examining the influence of changes in vessel diameter, stenosis length, and cross-sectional shape. The geometry model is shown in Figure 1 and detail dimensions for analyzed cases are summarized in Table 1. For artery size analysis, the base model taken from the previous article for stenosis with ${\left(A_{c}/A_{0}\right)}^{0.5}=0.4$ [10] was scaled proportionally in three dimensions in the 50–125% range. However, in the case of the throat length analysis, the length of the throat was changed in the range of 4 to 16 mm. In addition, in the case of the shape analysis of the influence of changes in the shape of the throat in the cross-section of the throat, the baseline model had ${\left(A_{c}/A_{0}\right)}^{0.5}=0.3$ [10], and the shape changes were obtained by asymmetric neck scaling in two directions to maintain a constant cross-sectional area. The prepared geometries were then used to conduct a detailed analysis of the impact of these three shape parameters on the maximum shear stresses and the hemolysis value. The obtained results were presented in graphs, and the tables with the results are available in Appendix A (Table A1, Table A2, Table A3, Table A4, Table A5 and Table A6).

**Figure 2:**
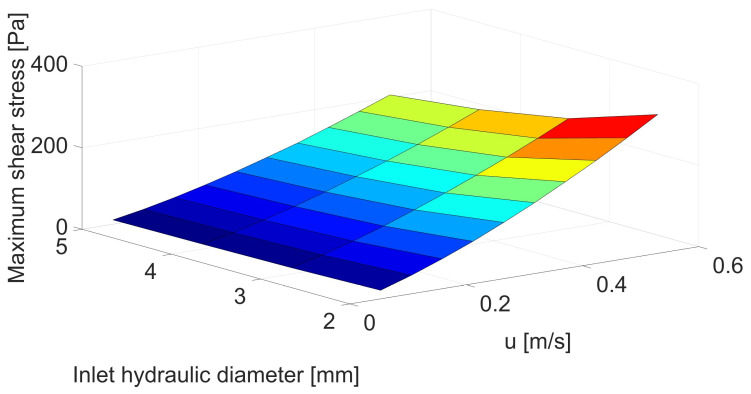
Correlation between inlet hydraulic diameter [mm] and mean velocity [m/s] for maximum shear stress [Pa].

Based on Figure 2 and Table A1, a strong correlation can be seen between vessel size and maximum shear stresses. The smaller the blood vessel, the greater the shear stress at the same linear velocity for the cross-section before the constriction. Consequently, the smaller the vessel, the lower the limiting velocity at which hemolysis of red blood cells can occur.

**Figure 3:**
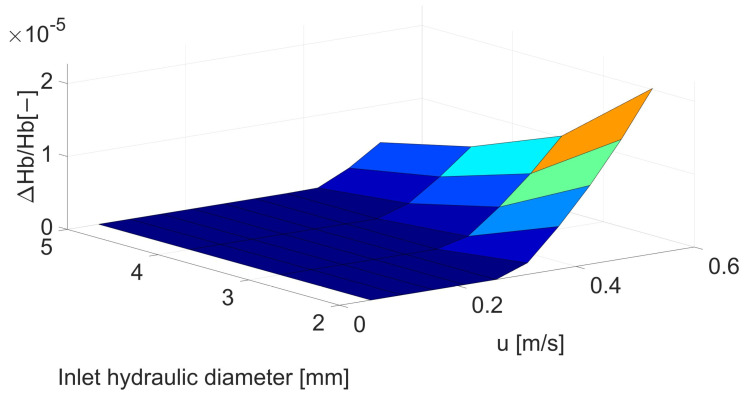
Correlation between inlet hydraulic diameter [mm] and mean velocity [m/s] for hemolysis [−].

Changes in maximum shear stresses are of crucial importance in assessing the risk of hemolysis; despite the increase in maximum stresses by about 50% (Figure 2 and Table A1) for the smallest artery at maximum flow, in the case of hemolysis, the difference reaches 193% (Figure 3 and Table A2) or even higher for lower velocities. This is related to both the increase in maximum stresses and the fluid volume in which the maximum shear stresses are exceeded, above which hemolysis is observed. 

**Figure 4:**
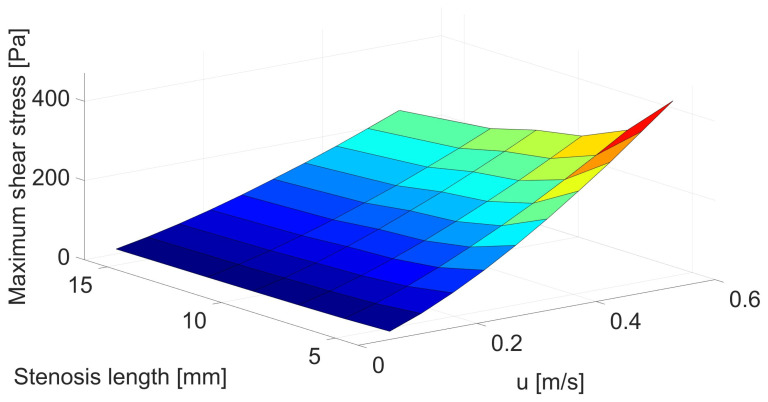
Correlation between stenosis length [mm] and mean velocity [m/s] for maximum shear stress [Pa].

Figure 4 and Table A2 show that the differences in maximum shear stresses are even more visible in the case of changes in the throat length. For the shortest stenosis, the increase in shear stress exceeds 100% compared to the base geometry with a stenosis length of 16 mm. Therefore, the shorter the throat, the greater the velocity acceleration and the greater the shear stress. Due to the rapid changes in velocity, apart from the increased risk of hemolysis, there is also a greater risk of damage to the vessel wall and the release of deposits, which can lead to embolism, which is very dangerous for the patient.

**Figure 5:**
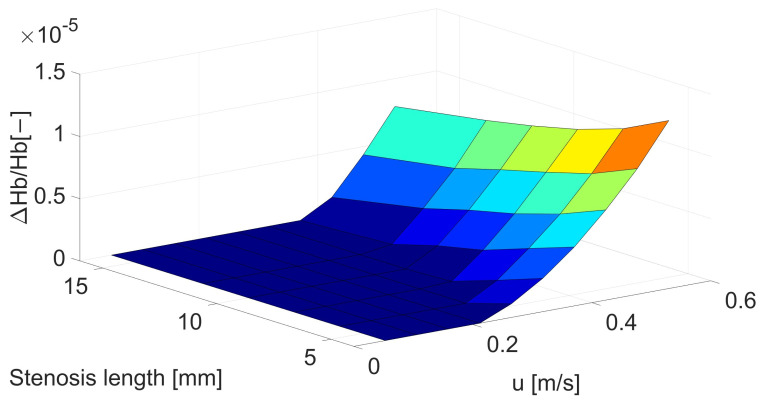
Correlation between stenosis length [mm] and mean velocity [m/s] for hemolysis [−].

As in the case of changes in the size of the artery, changes in the hemolysis value are also visible in the case of changes in the length of the stenosis. However, despite much more observable changes in shear stresses for changes in the narrowing length, the changes in the hemolysis value are not so large. In the case of maximum flow and minimum length, they reach 74% (Figure 5 and Table A4). However, these changes are much greater for lower velocities, which significantly reduces the critical velocity at which hemolysis begins.

**Figure 6:**
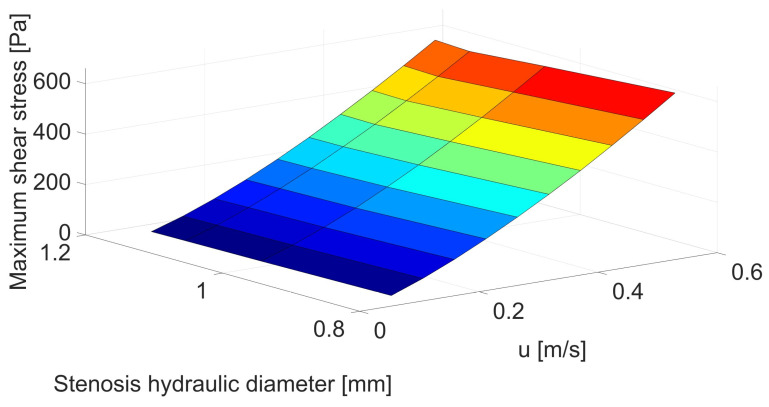
Correlation between stenosis hydraulic diameter [mm] and mean velocity [m/s] for maximum shear stress [Pa].

In contrast to the previous two shape parameters, the change in the shape of the cross-section has a much smaller impact on the maximum shear stresses, which can be seen both in Figure 6 and in the data from Table A5. Consequently, for most stenoses, the influence of this factor can be neglected unless the stenosis is very strongly flattened.

**Figure 7:**
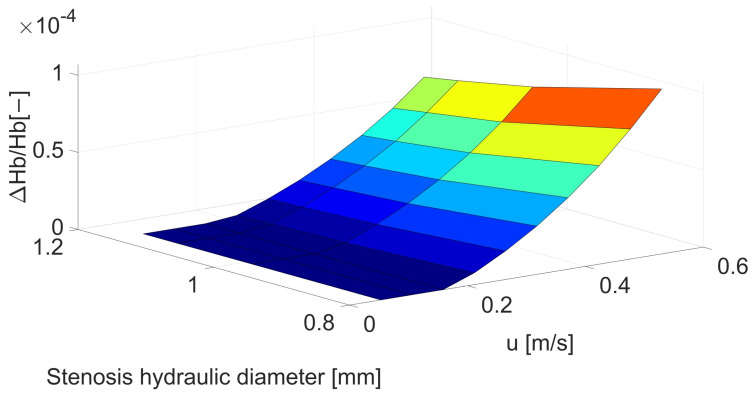
Correlation between stenosis hydraulic diameter [mm] and mean velocity [m/s] for hemolysis [−].

Despite slight differences in the maximum shear stresses observed in Figure 6, in the case of hemolysis, the differences are slightly larger, as seen in Figure 7 and Table A6. The increase in hemolysis reaches 49% for maximum flattening at maximum flow. However, in the case of lower speeds, the differences are more significant, similar to the previous issues. 

Based on the obtained results, it was decided to develop tables of correction factors for the equation for maximum shear stress. The resulting equation can pre-estimate the expected maximum shear stresses for a quick initial cholesteric stenosis analysis using typical medical data from non-invasive medical procedures. For this purpose, auxiliary dimensionless parameters for reading correction factors were introduced.

The first parameter is the size factor k. It is defined as the ratio of the size of the analyzed geometry x to the geometric dimensions of the base geometry X with a given degree of stenosis

(2) $$k=\frac{x}{X}\;.$$

The second parameter is the dimensionless stenosis length z, which is similarly defined as the ratio of the length of the analyzed stenosis l and the base model hydraulic diameter $D_{h0}$, taking into account the scale parameter

(3) $$z=\frac{l}{kD_{h0}}.$$

The last parameter is the dimensionless stenosis hydraulic diameter w, which is similarly defined as the ratio of the hydraulic diameter of the stenosis of the analyzed stenosis $d_{h}\;$ and the base model $D_{h}$, taking into account the scale parameter

(4) $$w=\frac{d_{h}\;}{kD_{h}}.$$

Considering the correction factors, the modified equation for the maximum shear stress is presented in Equation (5). However, the correction parameter related to the shape of the neck section can be omitted in most cases:

(5) $$\tau_{max}=\tau_{0}\cdot \beta_{1}\beta_{2}\beta_{3}≅\tau_{0}\cdot \beta_{1}\beta_{2}.$$

where $\tau_{max}\;$ is maximum shear stress in the analyzed case, $\tau_{0}$ is maximum shear stress for baseline artery stenosis geometry [10], and $\beta_{1}$, $\beta_{2}$, $\beta_{3}$ are correction coefficients. Correction factors are listed in Table 2, Table 3 and Table 4.

### 3.2. Enhanced Diagnostic Implementation for Internal Carotid Artery (ICA)

**Figure 8:**
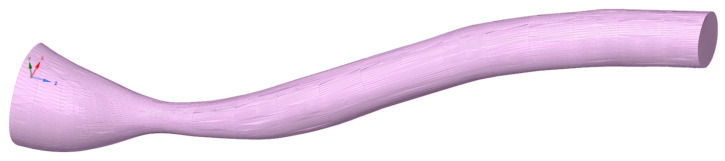
The geometry of internal carotid artery with stenosis.

The presented model has been tested in the context of application in the diagnosis of carotid artery stenoses. The geometry of the stenosis (Figure 8) was obtained as a result of the routine patient’s CT scans performed before endovascular treatment (angioplasty with stent placement). The hemolysis risk was checked at rest [33] and during physical exertion [34] corresponding to physical work or physical activity. Common carotid blood flow velocity data during graded exercise on a treadmill [34] were used and then recalculated to internal carotid artery (ICA) flow for physical activity conditions.

**Figure 9:**
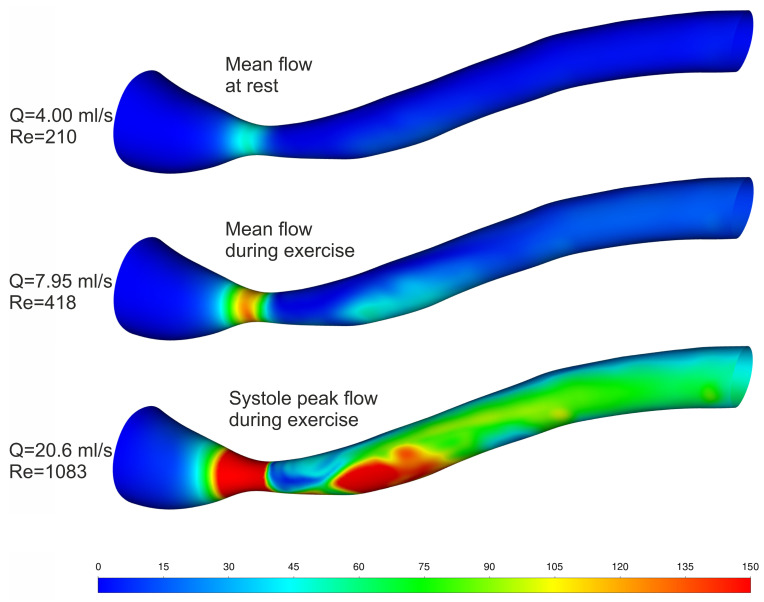
Wall shear stress [Pa] results for rest and exercise.

The geometry of the artery was obtained after geometry processing in MeshMixer and SpaceClaim to remove geometry distortions resulting from the finite resolution of the apparatus. The hydraulic diameter at the vessel inlet was 7.6 mm, and the distance between the beginning of the stenosis and the maximum stenosis was 10 mm. The measured degree of stenosis was ${\left(A_{c}/A_{0}\right)}^{0.5}=0.29$. The length of the entire geometry in the Z direction (Figure 9) was 50 mm. In addition, a fully developed velocity profile at the inlet to the stenotic carotid artery was assumed. Dimensionless geometry parameters were calculated according to Equations (2) and (3). Coefficients *β*_1_ and *β*_2_ were interpolated using spline function Matlab software (R2022b) with respect to velocities. Later, *β*_1_ was extrapolated to adjust the scale factor outside the previously considered region; however, power interpolation presented a high R^2^, higher than 0.99 for the analyzed region. In the case of *β*_2_, another spline interpolation with dimensionless stenosis length was in the table data range. All results are summed up in Table 5.

The mean velocity in the artery before the stenosis was obtained from the Fluent software (2023 R1). The maximum shear stresses for the base model $\tau_{0}$ were then calculated based on Equation (6) introduced in the previous article [10]:

(6) $$\tau_{0}=\alpha \cdot u^{\beta }\cdot {\left[{\left(\frac{A_{c}}{A_{0}}\right)}^{0.5}\right]}^{\gamma },$$

where α=24.21, β=1.346, γ=−3.385, and u stands for velocity before stenosis.

The correction factor β_1_ has a small value due to the high value of the scale factor k because the diameter of the vessel before the stenosis has a large diameter resulting from the presence of an aneurysm. On the other hand, the correction factor β_2_ is greater than unity because the obtained value of the dimensionless stenosis length z is slightly smaller than for the scaled base model. And, this shape parameter was calculated by comparing the distance between the beginning of the necking and the maximum necking due to the large differences in diameters before and after the stenosis. Based on the maximum shear stresses from the correlation $\tau_{0}$ for the base model and knowing the correction factors, the expected maximum stresses $\tau_{max}$ were calculated. The results obtained from the correlation were compared with the data obtained from the CFD simulation. In the case of flow at rest, the error was several percent; however, in the case of blood flow during exercise, this error decreased to about 10% in the case of average flow and a few percent in the case of maximum flow.

**Figure 10:**
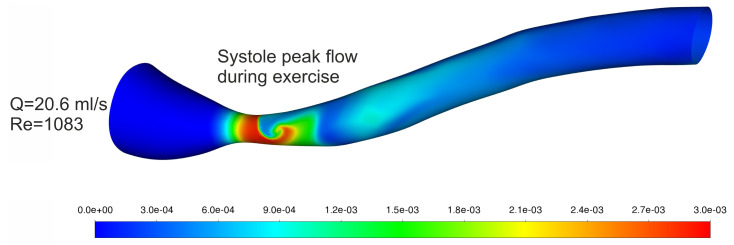
Hemolysis [−] results for exercise for systole peak blood flow.

The wall shear stresses are presented in Figure 9 to highlight the difference between rest and exercise conditions and also to present the areas where shear stress exceeds the shear threshold above which hemolysis occurs. It can be seen that increased blood flow is in line with increased wall shear stress. It is worth mentioning that, for mean flow, even during exercise, wall shear stresses are lower than 150 Pa [35,36,37,38], but during systole peak, there is a vast area where hemolysis may occur. The presented results are supplemented by Figure 10, where the hemolysis value can be seen. The largest fraction of hemolyzed blood cells can be observed in the constriction, where the greatest shear stresses occur. 

**Figure 11:**
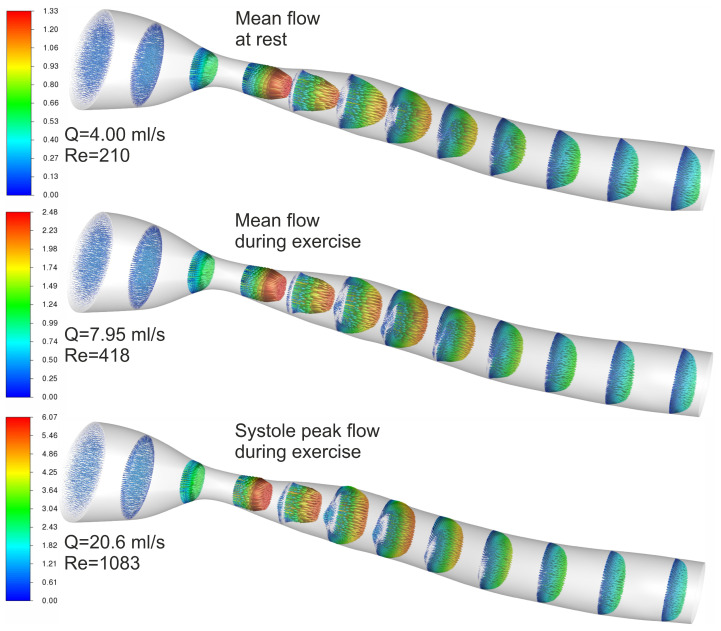
3D velocity magnitude [m/s] profile results for rest and exercise.

The velocity vectors presented in Figure 11 show that, in the stenosis region, velocity increases rapidly, forming a jet structure. The velocity profile in the stenosis throat is more flattened for higher blood flows. The stagnation zone is also visible and increases with higher blood flow.

## 4. Discussion

Cardiovascular diseases, including atherosclerosis, affect more patients [1,2,3]. Therefore, developing prevention and early diagnosis is increasingly important to protect patients’ health. Over the years, a number of methods for imaging cholesterol stenoses, such as optical coherence tomography [39,40,41,42,43], near infrared spectroscopy [44], ultrasound imaging [45,46,47,48], computed tomography [49,50,51,52,53,54,55], and magnetic resonance imaging [55,56,57,58,59,60,61], have been developed [62]. The information obtained thanks to imaging methods allowed for the visualization of stenoses and the creation of accurate CFD simulation models. CFD simulations can be a valuable tool in a wide range of blood flows in the circulatory system [10,27,63,64,65,66,67,68,69,70,71,72,73], providing valuable information for physicians. CFD can provide information on high shear stress [10,63,64,65], which is one of the factors causing blood hemolysis, which negatively affects patients’ health [74]. In addition, computational methods allow for the improvement of stents [75,76,77,78,79,80,81,82] used in PCI, or allow one to obtain “virtual” results of FFR measurements [83,84,85,86,87,88]. A wide range of computer simulation methods allows for extensive support of medics. However, there is room for tools for quick initial diagnostics before the more sophisticated tools offered by today’s computers are used. The presented model fills the gap, allowing for an instant preliminary hemolysis risk assessment based on non-invasive imaging methods and blood flow velocity. In addition, it can be implemented in software commonly used in hospitals, and artificial intelligence [61,89,90,91] can be responsible for identifying narrowing areas and their geometric parameters [92,93].

This study analyzed three shape parameters regarding their influence on the increase in maximum shear stresses and hemolysis. A strong correlation was observed between a decrease in vessel size and an increase in maximum shear stresses. There was a clear decrease in the critical velocity at which hemolysis occurs with a reduction in the diameter of the vessel. The increase in stress was largely reflected in the increase in hemolysis observed for smaller vessels. A similar rise in maximum shear stresses was observed for shorter constrictions, where the change in local velocity was faster. Stenosis with a sharp decrease in the vessel’s lumen is particularly dangerous due to the high stresses acting on the tissue, which may cause the uncontrolled release of cholesterol deposits, causing embolism. Stenoses of this shape are particularly dangerous during physical activity, resulting from physical work and sports. Patients exposed to such cholesterol changes are often still professionally active. Therefore, rapid diagnosis is very important. As far as hemolysis is concerned, the increase in shear stress observed for shorter constrictions translates into an increase in hemolysis. However, the increase is not as large as in the case of a decrease in size. This is due to a different distribution of shear stresses. The last parameter of the shape was the degree of flattening of the vessel in the narrowing, which translated into a decrease in the hydraulic diameter. In the case of shear stresses, an increase in shear stresses is observed only for strongly flattened narrowing, which is rarely observed. As in the other cases, the increase in shear stress translates into an increase in hemolysis. However, this increase is smaller than in the other analyzed cases. Based on the obtained results, Table 2, Table 3 and Table 4 present correction factors for the correlation, allowing us to estimate the maximum shear stresses presented in the previous article [10]. The obtained correlation was tested on the internal carotid artery stenosis and compared with the results of the CFD simulation; the error between the equation and the full simulation did not exceed a dozen or so percent and decreased with the increase in flow; for the maximum flow during exercise, it was only a few percent. The CFD simulations for the internal carotid artery show that increased blood flow leads to higher hemolysis, hence the need for prompt treatment, especially in physically active people, as they are more exposed to hemolysis of blood in the area of constrictions.

## 5. Conclusions

A helpful correlation was established between maximum shear stresses, a significant indicator of hemolysis risk, and blood flow and stenosis geometry. It has been shown that rapid changes in the cross-section of the vessel cause a greater risk of hemolysis than the same narrowing with a mild change in diameter on a larger section of the artery. Moreover, it has been shown that with a reduction in the vessel diameter, the critical flow velocity above which the risk of hemolysis occurs also decreases. The obtained correlation was tested on the actual geometry of a carotid atherosclerosis patient. The obtained results can be used to develop new guidelines for diagnosing atherosclerosis as a tool for a quick initial assessment of the patient’s condition.

## Figures and Tables

**Table 1:** Dimensions of analyzed geometry cases.

Case Number	D[mm]	$d_{h}[mm]$	$d_{h0}[mm]$	l[mm]	${\left(A_{c}/A_{0}\right)}^{0.5}[-]$
**1**	2	0.77	1.92	8	0.4
**2**	3	1.15	2.87	12	0.4
**3**	4	1.53	3.83	16	0.4
**4**	5	1.92	4.79	20	0.4
**5**	4	1.53	3.83	4	0.4
**6**	4	1.53	3.83	6	0.4
**7**	4	1.53	3.83	8	0.4
**8**	4	1.53	3.83	10	0.4
**9**	4	1.53	3.83	12	0.4
**10**	4	0.80	3.83	16	0.3
**11**	4	0.99	3.83	16	0.3
**12**	4	1.10	3.83	16	0.3
**13**	4	1.15	3.83	16	0.3

**Table 2:** List of *β*_1_ correction coefficients related to scale factor.

Velocity before Stenosis [m/s]	Scale Factor [−]			
	0.50	0.75	1.00	1.25
	β_1_ [−]			
**0.053**	1.68	1.24	1.00	0.85
**0.106**	1.61	1.21	1.00	0.86
**0.159**	1.58	1.20	1.00	0.87
**0.212**	1.54	1.19	1.00	0.88
**0.265**	1.52	1.19	1.00	0.88
**0.318**	1.51	1.18	1.00	0.88
**0.371**	1.50	1.18	1.00	0.88
**0.424**	1.49	1.18	1.00	0.89
**0.476**	1.48	1.17	1.00	0.89
**0.529**	1.47	1.17	1.00	0.89

**Table 3:** List of *β*_2_ correction coefficients related to dimensionless stenosis length.

Velocity before Stenosis [m/s]	Dimensionless Stenosis Length [−]					
	1.04	1.57	2.09	2.61	3.13	4.17
	β_2_ [−]					
**0.053**	1.65	1.34	1.21	1.13	1.08	1.00
**0.106**	1.81	1.43	1.26	1.16	1.10	1.00
**0.159**	1.90	1.48	1.29	1.21	1.11	1.00
**0.212**	1.94	1.51	1.31	1.22	1.10	1.00
**0.265**	1.98	1.53	1.32	1.23	1.10	1.00
**0.318**	2.00	1.54	1.32	1.24	1.10	1.00
**0.371**	2.02	1.55	1.33	1.25	1.11	1.00
**0.424**	2.03	1.56	1.33	1.25	1.11	1.00
**0.476**	2.04	1.56	1.34	1.25	1.11	1.00
**0.529**	2.04	1.56	1.34	1.25	1.11	1.00

**Table 4:** List of *β*_3_ correction coefficients related to dimensionless stenosis hydraulic diameter.

Velocity before Stenosis [m/s]	Dimensionless Stenosis Hydraulic Diameter [−]			
	0.70	0.86	0.96	1.00
	β_3_ [−]			
**0.053**	1.36	1.13	1.05	1.00
**0.106**	1.25	1.09	1.03	1.00
**0.159**	1.20	1.07	1.03	1.00
**0.212**	1.17	1.05	1.02	1.00
**0.265**	1.15	1.05	1.01	1.00
**0.318**	1.13	1.04	1.00	1.00
**0.371**	1.11	1.04	1.00	1.00
**0.424**	1.10	1.03	0.99	1.00
**0.476**	1.10	1.03	0.99	1.00
**0.529**	1.09	1.03	0.99	1.00

**Table 5:** Comparison of correlation and CFD results.

	Blood Flow [mL/s]	Velocity before Stenosis [m/s]	$\tau_{0}$ [Pa]	β_1_ [−]	β_2_ [−]	$\tau_{max}$ [Pa]	$\tau_{CFD}$ [Pa]	$\frac{\tau_{max}-\tau_{CFD}}{\tau_{CFD}}$ [%]
**Mean Flow at Rest**	4.00	0.088	60.3	0.6179	1.1397	42.5	51.6	−17.6
**Mean Flow** **during** **Exercise**	7.95	0.174	152.0	0.6436	1.2125	118.7	132.0	−10.1
**Systole Peak Flow during** **Exercise**	20.6	0.451	548.1	0.6829	1.2456	466.2	496.7	−6.1

**Figure A1:**
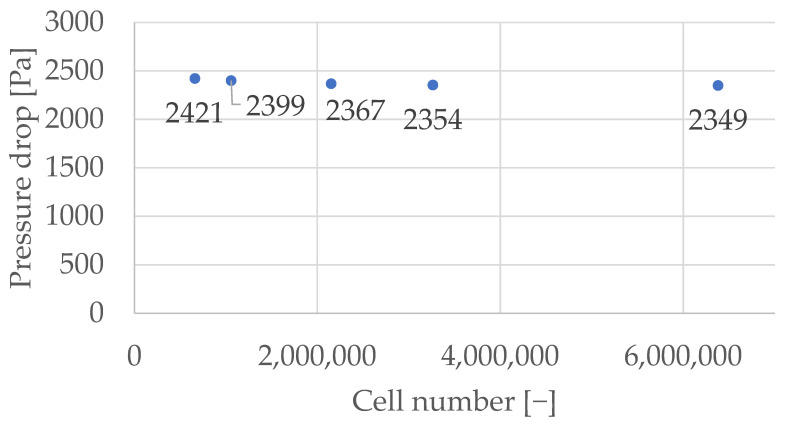
Grid independence study.

## Data Availability

Data will be made available on reasonable request.

## Appendix A

The main equation describing the behavior of fluids during flow is the Navier–Stokes equation. Its general form for an incompressible fluid is presented below.

(A1) $$\frac{\partial u}{\partial t}+\left(u\cdot \nabla \right)u-\nu \nabla^{2}u=-\nabla \left(\frac{p}{\rho }\right)+g\;$$

where ν is kinematic viscosity.

For laminar flows, this equation is sufficient for solving the flow field. However, for transient and turbulent flows, additional equations are needed due to the presence of time-varying turbulence affecting the flow field. A common solution to this problem is to average the Navier–Stokes equations based on Reynolds decompositions. In the Cartesian coordinates system, the Reynolds-averaged Navier–Stokes equations are as follows:

(A2) $$\rho {\bar{u}}_{j}\frac{\partial {\bar{u}}_{i}}{\partial x_{j}}=\rho {\bar{f}}_{i}+\frac{\partial }{\partial x_{j}}\left[-\bar{p}\delta_{ij}+\mu \left(\frac{\partial {\bar{u}}_{i}}{\partial x_{j}}+\frac{\partial {\bar{u}}_{j}}{\partial x_{i}}\right)-\rho \bar{u_{i}^{’}u_{j}^{’}}\right].\;$$

In addition to the RANS equation, equations describing turbulence are added to close the system of equations. The two most popular models using this approach are k−ϵ and k−ω and their derivatives.

Due to the complicated rheology of blood, it was necessary to use a non-Newtonian blood model. It was decided to use the PBBR model, which uses population balance to reflect the physiological processes of the agglomeration and deagglomeration of blood cells, which influence the change in the apparent fraction of red blood cell agglomerates in the cross-section of the artery.

The viscosity model used in this article can be described as follows:

(A3) $$\mu =M\left(\phi \right)\mu_{0}\;$$

where $\mu_{0}=6.99\;\cdot \;10^{-4}$ Pa·s is the viscosity obtained by fitting viscosity experimental data.

Equation (A4) presents the relative blood viscosity M(ϕ):

(A4) $$M\left(\phi \right)=\frac{M_{0}\left(\phi \right)+PeM_{\infty }\left(\phi \right)}{1+Pe}\;$$

And, Equation (A5) shows the Peclet number formula:

(A5) $$Pe=\frac{\dot{\gamma }a^{2}}{D_{M}\left(\phi \right)}\;$$

Furthermore, the relative blood viscosity for high shear rates $M_{\infty }(\phi )$ is defined as follows:

(A6) $$M_{\infty }\left(\phi \right)={\left(1-\phi \right)}^{-5/2}-C\left\{\ln\left[1-{\left(\frac{\phi }{\phi^{*}}\right)}^{1/3}\right]+\sum_{j=1}^{6}\frac{1}{j}{\left(\frac{\phi }{\phi^{*}}\right)}^{j/3}\right\}\;$$

And, the relative blood viscosity for low shear rates $M_{0}(\phi )$ is defined in Equation (A7):

(A7) $$M_{0}\left(\phi \right)={\left(1-\phi \right)}^{-5/2}+1.3\left[{\left(1-\frac{\phi }{\phi^{*}}\right)}^{-2}-\sum_{j=0}^{2}\left(1+j\right){\left(\frac{\phi }{\phi^{*}}\right)}^{j}\right]\;$$

where $\dot{\gamma }$ is the shear rate, a is the characteristic particle size for the whole population of agglomerates and is equal to the volume-weighted mean particle size, ϕ is the effective apparent volume fraction of red blood cells, $D_{M}(\varphi )$ is the effective diffusion coefficient [8] for molecular diffusion, C ≅ 2 and is an equation constant, and $\phi^{*}$ is the maximum apparent volume fraction equal to 0.695 [8,94,95].

Furthermore, the hemolysis model was presented in detail in previous publications [8,10,27]; however, the main equation describing hemolysis is as follows:

(A8) $$\frac{\partial H_{L}}{\partial t}+\frac{\partial }{\partial x_{i}}\left({\bar{u}}_{i}H_{L}\right)-\frac{\partial }{\partial x_{i}}\left(D_{EFF}\frac{\partial H_{L}}{\partial x_{i}}\right)=\delta A\tau^{\theta }\left(1-H_{L}\right)\;$$

where $D_{EFF}$ is the effective diffusion coefficient for molecular and turbulent diffusion and

(A9) $$\delta =\left\{\begin{array}{c} 0,\;if\;\tau <\tau_{s}\\ 1,\;if\;\tau \geq \tau_{s} \end{array}\right..$$

Moreover, based on various articles [35,36,37,38], the value of shear threshold $\tau_{s}$ was set to 150 Pa.

The numerical mesh was made in Fluent Meshing. A polyhedral mesh with a boundary layer was generated. A mesh independence test was performed for the carotid artery model for mean exercise blood flow for five different meshes ranging from several hundred thousand elements to several million cells. The results of the mesh independence test are presented in Figure A1, where the pressure drop is compared depending on the mesh size. A mesh with 2.15 million cells was chosen because the difference in pressure drop compared to the densest mesh was below 1%. The final mesh had a maximum cell size set to 0.2 mm, and the boundary layer was generated with a uniform algorithm for 12 layers with a growth rate of 1.2 and a first layer height of 0.01 mm.

The tables summarize the results of CFD calculations presented in Figure 2, Figure 3, Figure 4, Figure 5, Figure 6 and Figure 7.

**Table A1:** Maximum shear stress [Pa] vs. inlet hydraulic diameter [mm].

Inlet Velocity [m/s]	Velocity before Stenosis [m/s]	Inlet Hydraulic Diameter [mm]			
		2	3	4	5
		Maximum Shear Stress [Pa]			
0.05	0.053	2.09 ⋅10	1.53 ⋅10	1.24 ⋅10	1.06 ⋅10
0.10	0.106	4.54 ⋅10	3.42 ⋅10	2.81 ⋅10	2.43 ⋅10
0.15	0.159	7.34 ⋅10	5.59 ⋅10	4.66 ⋅10	4.07 ⋅10
0.20	0.212	1.04$\;\cdot 10^{2}$	8.05 ⋅10	6.76 ⋅10	5.93 ⋅10
0.25	0.265	1.38$\;\cdot 10^{2}$	1.08$\;\cdot 10^{2}$	9.07 ⋅10	7.97 ⋅10
0.30	0.318	1.74$\;\cdot 10^{2}$	1.37$\;\cdot 10^{2}$	1.16$\;\cdot 10^{2}$	1.02$\;\cdot 10^{2}$
0.35	0.371	2.13$\;\cdot 10^{2}$	1.68$\;\cdot 10^{2}$	1.42$\;\cdot 10^{2}$	1.25$\;\cdot 10^{2}$
0.40	0.424	2.53$\;\cdot 10^{2}$	2.00$\;\cdot 10^{2}$	1.70$\;\cdot 10^{2}$	1.51$\;\cdot 10^{2}$
0.45	0.476	2.96$\;\cdot 10^{2}$	2.34$\;\cdot 10^{2}$	2.00$\;\cdot 10^{2}$	1.78$\;\cdot 10^{2}$
0.50	0.529	3.41$\;\cdot 10^{2}$	2.70$\;\cdot 10^{2}$	2.31$\;\cdot 10^{2}$	2.06$\;\cdot 10^{2}$

**Table A2:** ΔH_b_/H_b_ [−] vs. inlet hydraulic diameter [mm].

Inlet Velocity [m/s]	Velocity before Stenosis [m/s]	Inlet Hydraulic Diameter [mm]			
		2	3	4	5
		ΔH_b_/H_b_ [−]			
0.05	0.053	0	0	0	0
0.10	0.106	0	0	0	0
0.15	0.159	0	0	0	0
0.20	0.212	0	0	0	0
0.25	0.265	0	0	0	0
0.30	0.318	1.57$\;\cdot 10^{-6}$	0	0	0.
0.35	0.371	5.87$\;\cdot 10^{-6}$	1.06$\;\cdot 10^{-6}$	0	0.
0.40	0.424	1.08$\;\cdot 10^{-5}$	4.37$\;\cdot 10^{-6}$	1.39$\;\cdot 10^{-6}$	5.78$\;\cdot 10^{-8}$
0.45	0.476	1.64$\;\cdot 10^{-5}$	8.24$\;\cdot 10^{-6}$	4.35$\;\cdot 10^{-6}$	2.08$\;\cdot 10^{-6}$
0.50	0.529	2.27$\;\cdot 10^{-5}$	1.27$\;\cdot 10^{-5}$	7.76$\;\cdot 10^{-6}$	4.88$\;\cdot 10^{-6}$

**Table A3:** Maximum shear stress [Pa] vs. stenosis length [mm].

Inlet Velocity [m/s]	Velocity before Stenosis [m/s]	Stenosis Length [mm]					
		4	6	8	10	12	16
		Maximum Shear Stress [Pa]					
0.05	0.053	2.04 ⋅10	1.67 ⋅10	1.50 ⋅10	1.40 ⋅10	1.33 ⋅10	1.24 ⋅10
0.10	0.106	5.09 ⋅10	4.02 ⋅10	3.55 ⋅10	3.28 ⋅10	3.08 ⋅10	2.81 ⋅10
0.15	0.159	8.84 ⋅10	6.91 ⋅10	6.02 ⋅10	5.62 ⋅10	5.15 ⋅10	4.66 ⋅10
0.20	0.212	1.31$\;\cdot 10^{2}$	1.02$\;\cdot 10^{2}$	8.83 ⋅10	8.27 ⋅10	7.47 ⋅10	6.76 ⋅10
0.25	0.265	1.79$\;\cdot 10^{2}$	1.39$\;\cdot 10^{2}$	1.19$\;\cdot 10^{2}$	1.12$\;\cdot 10^{2}$	1.00$\;\cdot 10^{2}$	9.07 ⋅10
0.30	0.318	2.31$\;\cdot 10^{2}$	1.78$\;\cdot 10^{2}$	1.53$\;\cdot 10^{2}$	1.44$\;\cdot 10^{2}$	1.28$\;\cdot 10^{2}$	1.16$\;\cdot 10^{2}$
0.35	0.371	2.87$\;\cdot 10^{2}$	2.20$\;\cdot 10^{2}$	1.89$\;\cdot 10^{2}$	1.77$\;\cdot 10^{2}$	1.57$\;\cdot 10^{2}$	1.42$\;\cdot 10^{2}$
0.40	0.424	3.45$\;\cdot 10^{2}$	2.6$\;\cdot 10^{2}$	2.27$\;\cdot 10^{2}$	2.13$\;\cdot 10^{2}$	1.89$\;\cdot 10^{2}$	1.70$\;\cdot 10^{2}$
0.45	0.476	4.08$\;\cdot 10^{2}$	3.12$\;\cdot 10^{2}$	2.68$\;\cdot 10^{2}$	2.50$\;\cdot 10^{2}$	2.22$\;\cdot 10^{2}$	2.00$\;\cdot 10^{2}$
0.50	0.529	4.71$\;\cdot 10^{2}$	3.61$\;\cdot 10^{2}$	3.10$\;\cdot 10^{2}$	2.89$\;\cdot 10^{2}$	2.57$\;\cdot 10^{2}$	2.31$\;\cdot 10^{2}$

**Table A4:** ΔH_b_/H_b_ [−] vs. stenosis length [mm].

Inlet Velocity [m/s]	Velocity before Stenosis [m/s]	Stenosis Length [mm]					
		4	6	8	10	12	16
		ΔH_b_/H_b_ [−]					
0.05	0.053	0	0	0	0	0	0
0.10	0.106	0	0	0	0	0	0
0.15	0.159	0	0	0	0	0	0
0.20	0.212	0	0	0	0	0	0
0.25	0.265	1.09$\;\cdot 10^{-6}$	0	0	0	0	0
0.30	0.318	2.68$\;\cdot 10^{-6}$	1.38$\;\cdot 10^{-6}$	1.45$\;\cdot 10^{-7}$	0	0	0
0.35	0.371	4.73$\;\cdot 10^{-6}$	3.47$\;\cdot 10^{-6}$	2.19$\;\cdot 10^{-6}$	1.40$\;\cdot 10^{-6}$	3.67$\;\cdot 10^{-7}$	0
0.40	0.424	7.25$\;\cdot 10^{-6}$	5.77$\;\cdot 10^{-6}$	4.65$\;\cdot 10^{-6}$	3.80$\;\cdot 10^{-6}$	2.79$\;\cdot 10^{-6}$	1.39$\;\cdot 10^{-6}$
0.45	0.476	1.01$\;\cdot 10^{-5}$	8.55$\;\cdot 10^{-6}$	7.52$\;\cdot 10^{-6}$	6.58$\;\cdot 10^{-6}$	5.55$\;\cdot 10^{-6}$	4.35$\;\cdot 10^{-6}$
0.50	0.529	1.35$\;\cdot 10^{-5}$	1.17$\;\cdot 10^{-5}$	1.05$\;\cdot 10^{-5}$	9.64$\;\cdot 10^{-6}$	8.93$\;\cdot 10^{-6}$	7.76$\;\cdot 10^{-6}$

**Table A5:** Maximum shear stress [Pa] vs. stenosis hydraulic diameter [mm].

Inlet Velocity [m/s]	Velocity before Stenosis [m/s]	Stenosis Hydraulic Diameter [mm]			
		0.80	0.99	1.10	1.15
		Maximum Shear Stress [Pa]			
0.05	0.053	4.16 ⋅10	3.46 ⋅10	3.19 ⋅10	3.05 ⋅10
0.10	0.106	8.91 ⋅10	7.77 ⋅10	7.36 ⋅10	7.13 ⋅10
0.15	0.159	1.44$\;\cdot 10^{2}$	1.28$\;\cdot 10^{2}$	1.23$\;\cdot 10^{2}$	1.20$\;\cdot 10^{2}$
0.20	0.212	2.05$\;\cdot 10^{2}$	1.84$\;\cdot 10^{2}$	1.78$\;\cdot 10^{2}$	1.75$\;\cdot 10^{2}$
0.25	0.265	2.71$\;\cdot 10^{2}$	2.47$\;\cdot 10^{2}$	2.38$\;\cdot 10^{2}$	2.36$\;\cdot 10^{2}$
0.30	0.318	3.41$\;\cdot 10^{2}$	3.14$\;\cdot 10^{2}$	3.03$\;\cdot 10^{2}$	3.02$\;\cdot 10^{2}$
0.35	0.371	4.15$\;\cdot 10^{2}$	3.86$\;\cdot 10^{2}$	3.71$\;\cdot 10^{2}$	3.73$\;\cdot 10^{2}$
0.40	0.424	4.94$\;\cdot 10^{2}$	4.61$\;\cdot 10^{2}$	4.44$\;\cdot 10^{2}$	4.47$\;\cdot 10^{2}$
0.45	0.476	5.76$\;\cdot 10^{2}$	5.41$\;\cdot 10^{2}$	5.20$\;\cdot 10^{2}$	5.25$\;\cdot 10^{2}$
0.50	0.529	6.61$\;\cdot 10^{2}$	6.23$\;\cdot 10^{2}$	5.99$\;\cdot 10^{2}$	6.06$\;\cdot 10^{2}$

**Table A6:** ΔH_b_/H_b_ [−] vs. stenosis hydraulic diameter [mm].

Inlet Velocity [m/s]	Velocity before Stenosis [m/s]	Stenosis Hydraulic Diameter [mm]			
		0.80	0.99	1.10	1.15
		ΔH_b_/H_b_ [−]			
0.05	0.053	0	0	0	0
0.10	0.106	0	0	0	0
0.15	0.159	0	0	0	0
0.20	0.212	7.59$\;\cdot 10^{-6}$	3.84$\;\cdot 10^{-6}$	2.46$\;\cdot 10^{-6}$	2.05$\;\cdot 10^{-6}$
0.25	0.265	1.85$\;\cdot 10^{-5}$	1.25$\;\cdot 10^{-5}$	1.02$\;\cdot 10^{-5}$	9.45$\;\cdot 10^{-6}$
0.30	0.318	3.13$\;\cdot 10^{-5}$	2.27$\;\cdot 10^{-5}$	1.95$\;\cdot 10^{-5}$	1.82$\;\cdot 10^{-5}$
0.35	0.371	4.64$\;\cdot 10^{-5}$	3.48$\;\cdot 10^{-5}$	3.04$\;\cdot 10^{-5}$	2.86$\;\cdot 10^{-5}$
0.40	0.424	6.39$\;\cdot 10^{-5}$	4.89$\;\cdot 10^{-5}$	4.30$\;\cdot 10^{-5}$	4.07$\;\cdot 10^{-5}$
0.45	0.476	8.43$\;\cdot 10^{-5}$	6.56$\;\cdot 10^{-5}$	5.83$\;\cdot 10^{-5}$	5.50$\;\cdot 10^{-5}$
0.50	0.529	1.07$\;\cdot 10^{-4}$	8.50$\;\cdot 10^{-5}$	7.56$\;\cdot 10^{-5}$	7.18$\;\cdot 10^{-5}$
